# Peptide-based synthetic vaccines

**DOI:** 10.1039/c5sc03892h

**Published:** 2015-12-17

**Authors:** Mariusz Skwarczynski, Istvan Toth

**Affiliations:** a The University of Queensland , School of Chemistry and Molecular Biosciences , St Lucia 4072 , Australia . Email: m.skwarczynski@uq.edu.au; b The University of Queensland , Institute for Molecular Bioscience , St Lucia 4072 , Australia; c The University of Queensland , School of Pharmacy , Brisbane , QLD 4072 , Australia

## Abstract

Classically all vaccines were produced using live or attenuated microorganisms or parts of them. However, the use of whole organisms, their components or the biological process for vaccine production has several weaknesses. The presence of immunologically redundant biological components or biological impurities in such vaccines might cause major problems. All the disadvantageous of traditional vaccines might be overcome *via* the development of fully synthetic peptide-based vaccines. However, once minimal antigenic epitopes only are applied for immunisation, the immune responses are poor. The use of an adjuvant can overcome this obstacle; however, it may raise new glitches. Here we briefly summarise the current stand on peptide-based vaccines, discuss epitope and adjuvant design, and multi-epitope and nanoparticle-based vaccine approaches. This mini review discusses also the disadvantages and benefits associated with peptide-based vaccines. It proposes possible methods to overcome the weaknesses of the synthetic vaccine strategy and suggests future directions for its development.

## Abstract 2

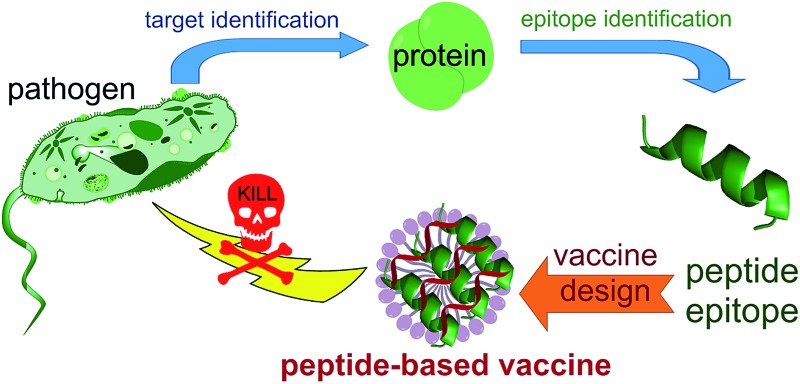
This review summarise the current stand and future perspective on synthetic peptide-based vaccines.

## Introduction

Vaccination is among the most successful medical treatments ever developed. This prophylaxis had a long journey through history to become one of humanity's key achievements; from early immunisation in China, centuries ago, through to Edward Jenner's works in the eighteenth century – when the word “*vaccination*” was introduced for the first time – up to these modern times when recombinant protein-based vaccines are increasingly becoming popular. Despite the advances in the field, classical vaccination using whole organisms is still common. Whole pathogen immunisations usually produce long lasting immunity; however, they are not without drawbacks. For example, the safety of this form of vaccination is one of the major concerns as it may cause autoimmune or strong allergic responses. Interestingly, allergic shock is often related not to the presence of pathogen itself but rather, it is caused by contamination from the medium on which microorganism was grown (*e.g.* eggs, antibiotics). Attenuation or inactivation of such vaccines might not be perfect and the pathogen may return to its virulent state. One of the most prominent examples of such vaccine defectiveness was the “Lübeck disaster”, when, in 1930, 67 babies among the 249 vaccinated with tuberculosis vaccine (BCG) died.^1^ Shedding of the pathogen to the environment, during vaccine manufacture, is the other problem and infections of staff during the production process have been also reported.^2^ Manufacturing difficulties of some pathogen (*e.g.* malaria sporozoites), poor vaccine stability and the need for a “cold chain” are other significant disadvantages of classical vaccines. Some of the vaccines cannot even use the whole cell approach (*e.g.* cancer vaccines, due to tumour similarity to healthy human cells). Subunit vaccines utilising only part of the whole pathogen are more controllable and can be produced without the use of the pathogen itself (*e.g.* recombinant proteins). They are a very attractive alternative to the whole pathogen approach and have become extensively popular in the modern era. However, they are still not perfectly safe, and cause side effects and production difficulties similar to whole pathogen strategies. For example whole protein-based approach was largely abandoned in the case of the vaccine against Group A *Streptococcus* which was targeting surface protein (M-protein) of the bacteria due to potential protein-triggered autoimmunity.^3^ In addition to problems associated with protein purities (these are normally produced using microorganisms), there are common stability issues, large scale protein expression difficulties, difficulties with the introduction of desired post-translational modification (*e.g.* glycosylation) into recombinant proteins and poor or undesired immune responses (inflammation, autoimmunity, *etc.*). Therefore, the use of only minimal antigenic epitopes which can trigger the desired immune responses appears to be the smart approach to develop safe vaccines. The synthetic peptide-based vaccines may have such a capacity. They may become the unique medication of the future capable of delivering not only protection against diseases but may turn into the therapeutic tool to treat them.

## Vaccination and immunity

**Fig. 1:**
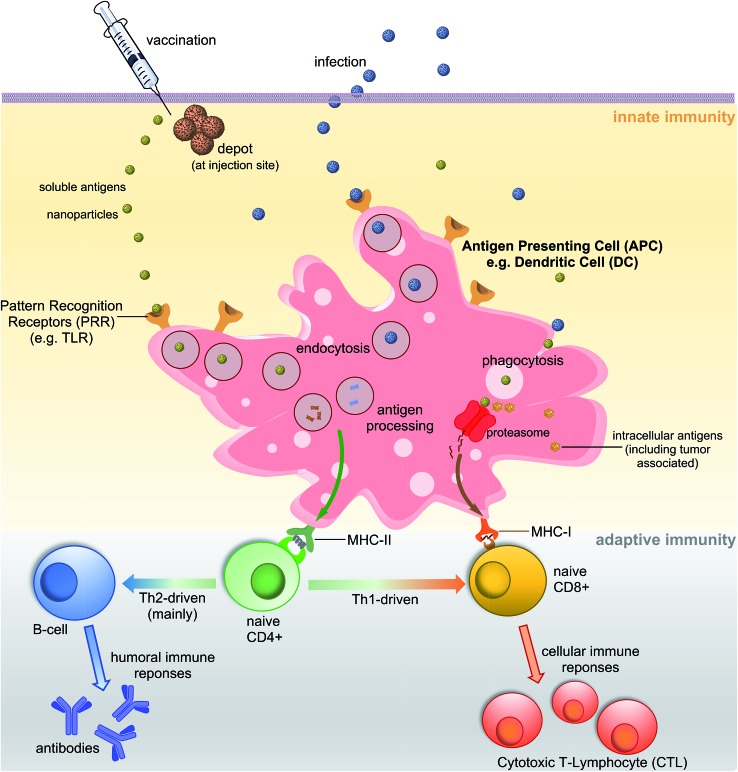
Schematic representation of major pathways of immune response.

A vaccine, similar to a natural pathogen, at first, needs to be recognised by an animal/human defence system as an “enemy” to trigger a cascade of immune responses (Fig. 1). The innate immune system serves as the first line of defence against microbial aggressors or toxins (produced by them). It also recognises pathogens/antigens as invaders and stimulates adaptive immunity, triggering antibodies and cellular responses. Antigen-presenting cells (APCs) such as dendritic cells (DCs) or macrophages are able to recognise pathogen-associated molecular patterns (PAMPs) *via* pattern recognition receptors (PRRs) such as toll-like receptors (TLRs). The PAMPs are recognised before or during the endocytosis process of an antigen by APCs. Once recognised, antigens are processed into small molecules (usually peptides) and loaded on MHC-I or MHC-II proteins.^4,5^ MHC-II loaded with small antigen trigger the activation of T-helper cells (CD4) which further activate cellular immunity (cytotoxic T-lymphocyte (CTL) responses) and/or humoral immunity (neutralising and/or opsonic antibodies production by B-cells). Antigens loaded on MHC-I interact directly with CD8+ cells stimulating cellular responses. Antigen can be recognised, processed and transported to lymph nodes by peripheral APCs, or it may travel on its own to lymphatic nodes and then be processed by lymph node resident APCs. Lymph nodes are composed mostly of T-cells, B-cells, DCs and macrophages, and one of the major sites for activation of adaptive immunity.^6^

One of the important characteristics of immune responses is the T-helper subtype activation and corresponding type-specific cytokines release. Antigen loaded on MHC-II can activate both Th1 and Th2-types helper cells. Th2 cells trigger mainly humoral responses against extracellular pathogens, while Th1 cells activate cellular immunity against intracellular pathogen (viruses, cancer). However, Th1 and Th2 are not strictly equal with cell-mediated and humoral immunity, respectively.^7^ For example, the Th1 pathway may also stimulate modest levels of antibody-based responses. Th1 cytokines tend to produce the pro-inflammatory responses while Th2 is associated with the anti-inflammatory responses. Imbalanced Th1/Th2 responses may cause immunopathological complications such as tissue damage *via* extensive inflammation or strong allergic responses.^8^ Thus, a properly-balanced Th1 and Th2 responses should be taken into account during the vaccine development process.

## Peptide-based vaccine

**Fig. 2:**
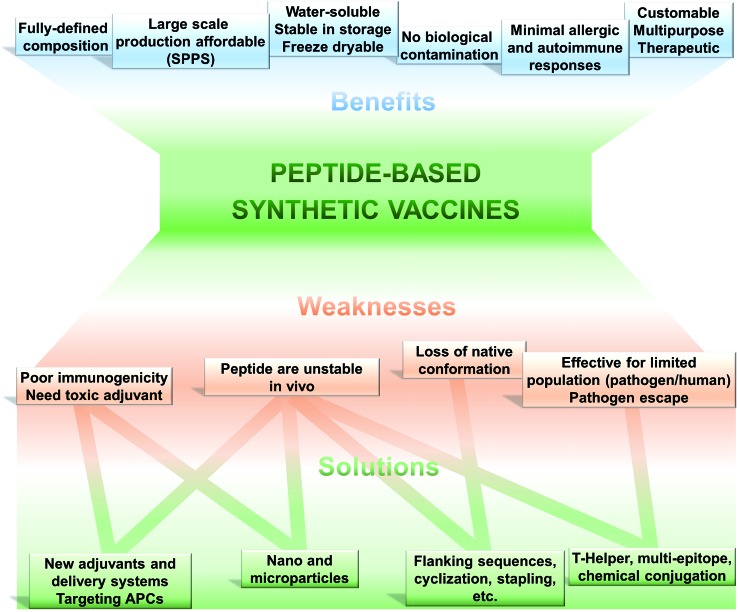
Peptide-based vaccines; pros, cons and solutions.

The use of only a minimal microbial component which is able to stimulate long lasting protection against the pathogen is becoming the tendency in vaccine development. Thus, fully synthetic peptide-based vaccines are the potential future of vaccination (Fig. 2). This type of vaccine may not replace the recent trend in development of recombinant protein-based vaccines in the near future; however, exciting development in peptide-based immunogens is already occurring.

The key feature of peptide based vaccines are as follow:^9–12^

(1) Peptide-based vaccines are produced almost exclusively using chemical synthetic approaches. Peptide antigen can be fully and precisely characterised as a chemical entity (analogously to classical drugs).

(2) Production of peptides becomes simple, easily reproducible, fast and cost-effective due to recent developments in solid phase peptide synthesis (SPPS) using automatic synthesisers and application of microwave techniques.

(3) Chemical synthesis practically removes all the problems associated with the biological contamination of the antigens.

(4) These vaccines are typically water-soluble, stable under simple storage conditions (generally does not require “cold chain”), can be freeze-dried, and their stability can be easily assessed using standard physicochemical characterisation methods.

(5) Peptides can be customised to target very specific objectives. The immune responses can be directed against naturally non-immunodominant epitopes. By the use of a multi-epitope approach, single peptide-based vaccine can be designed to target several strains, different stages of life cycle or even different pathogens.

(6) Peptide antigens are less likely to induce allergic or autoimmune responses due to the lack of redundant elements.

Such an approach to vaccination is not free from challenges to be overcome (Fig. 2). Peptides are very poor immunogens on their own and need assistance of adjuvants (immune stimulants) or at least a delivery system. They are very susceptible to enzymatic degradation, significantly more than folded protein. They are often not recognised equally by the whole outbred population, such as humans. However, this weakness is also suffered, to some extent, by other subunit vaccines (including recombinant protein-based vaccines).

## Epitope design

The choice of an epitope is a crucial step in the design of a peptide-based vaccine. Therefore, appropriate peptide epitopes on the protein of interest at first need to be identified. These epitopes should be able to induce strong, long-lasting humoral and/or cellular immunity against the desired pathogen. However, epitopes chosen for peptide vaccine design are not always the immune dominant epitopes against which humans predominantly induce immune responses. For example, antibodies from humans infected with hookworms recognize dominant epitope on *Necator americanus* APR-1 protein but do not offer any protection against hookworm.^13^ While other APR-1 epitope, poorly recognized by human upon natural infection, showed ability to induce production of neutralising antibodies. Therefore the latter non-dominant epitope was suggested as a promising candidate for peptide-based vaccine development.^13^ The selection of epitope also needs to take into account possible hypersensitivity responses associated with some of the antigens. Several IgE-inducing epitopes were reported to partially overlap with IgG epitopes in the Na-ASP-2 protein from hookworm and cause immediate-type hypersensitivity reactions after vaccination in humans.^14^ Finally, the chosen epitope needs to be highly conserved or a mixture of several epitopes will be required for vaccine to cover variety of pathogen subtypes.

**Fig. 3:**
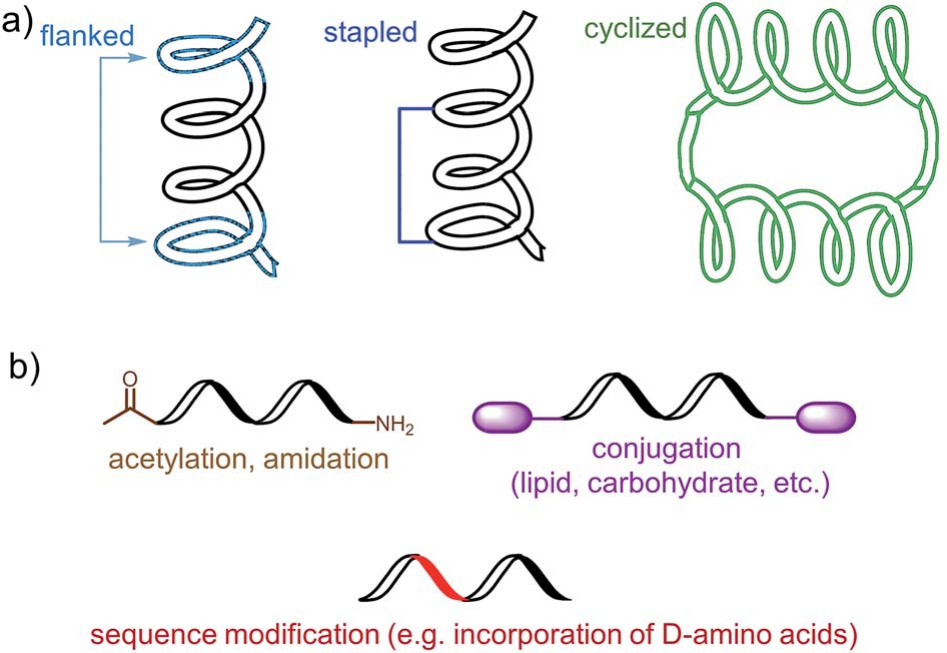
Examples of an epitope modification to (a) stabilize conformation (and improve stability against enzymatic degradation) and (b) improve its stability *in vivo*.

Most of B-cell epitopes required to induce the desired humoral immunity have to maintain their native conformation found in the protein. While the length of the minimum B-cell epitopes may significantly vary and starts from as few as five amino acids, they are incorporated into peptide-based vaccines as significantly longer peptides to maintain their native conformation which a short sequence could not adopt. Alternatively, to maintain proper conformation, short peptide epitopes can be flanked with sequences inducing the desired secondary structure (Fig. 3a). For example, Good and co-workers used sequences derived from yeast GCN4 protein to promote the desired conformation on the short peptides.^15^ This sequence was used to flank the B-cell epitopes on its C and N-terminus allowing them to form an α-helix. The antibodies raised against the resultant peptide were able to recognize the parent protein.

Stapled peptide is the other approach which allows the adoption of the desired conformation to shorter peptides (Fig. 3a). This strategy is based on introducing an “artificial” chemical bond between distinct side chains of amino acids, not only forcing the peptide to fold in the desired conformation but also to protect peptides against proteolytic degradation. It is important to notice that stapling the peptide should not alter the epitope recognition site. For example, Walensky and co-workers produced peptide-based antigens by stapling an epitope derived from HIV-1 gp41-protein which resulted in stabilisation of its α-helical structure, improved proteolytic stability, and its high affinity binding with neutralizing antibodies.^16^

Chemical modification of epitopes naturally requires careful examination whenever the amendment of the structure does not alter the desired peptide immunological properties.^17,18^ Thus one of the important advantages of peptide-based vaccines is their ability to preferentially stimulate an immune response against regions of the protein that are critical for pathogen functions but are not very immunogenic or easily accessible under normal conditions. Hodges and coworkers demonstrated that a peptide-based, but not the protein derived, vaccine was able to stimulate a high antibody titer against a native receptor-binding domain of pilin protein from *Pseudomonas aeruginosa*.^19^ Vaccine design based on prediction of the secondary structure of peptide antigen may fail to achieve desired efficacy by the forcing of peptide to adopt the wrong conformation, especially when the secondary structure of the protein antigen is not fully confirmed.

In contrast, to induce cellular immunity, conformational presentation of epitopes is not required. The CD8 epitope needs to be presented to MHC I proteins, after the processing of antigen, as a strictly defined linear sequence of eight to ten amino acids. Therefore processing longer peptides into shorter epitopes is crucial, but not the conformation of an epitope. These properties of CD8 epitopes allow the relatively easy computational prediction of the epitopes in the protein sequence but unfortunately create other difficulties. Modification of such epitopes toward conjugation into a delivery system or improving their solubility may diminish their immunogenicity. For example, when CD8 epitope from human papillomavirus (HPV) E7 protein was modified on its C-terminus, therapeutic antitumor potency of vaccine bearing the epitope was greatly reduced or even completely diminished.^20^

Human leukocyte antigen (HLA) genes encoding MHCs are exceedingly diverse among humans causing the situation that an epitope recognised by one MHC protein (and therefore one human population) might not be recognised by another. Therefore, knowledge on epitope recognition among the targeted human population is an important factor in peptide vaccine design. The additional difficulties in vaccine development rise when model research animals recognise different epitopes from the protein of interest than the majority of the human population.

T-Helper cells play a crucial role to link innate and adaptive immunity (Fig. 1) and T-helper epitopes are crucial components of peptide-based vaccine. While immune responses without the presence of T-helper are possible, they are weaker, uneven in heterogeneous population and memory responses are impaired. T-helper can be both disease-specific (derived from the protein of the targeted pathogen) and universal, such as an artificial pan-DR helper T-lymphocyte epitope (PADRE). This synthetic peptide was designed to bind most of the human HLA-DR receptors, providing “universal” immune stimulation in a heterogeneous population.^21^ While protein carrier can be also applied as a source of T-helper epitopes, such carrier may induce undesirable immune responses against itself. Thus, incorporation of universal T-helper epitopes in peptide-based vaccine has become a very successful approach since the discovery of these epitopes in the early 1990s.^22,23^

PADRE is also a good example of epitope engineered toward higher metabolic stability. The epitope includes d-alanine, l-cyclo-hexylalanine and amino-caproic acid residues which greatly improve its stability against enzymatic degradation. There are also other methods to improve metabolic stability of peptide antigens, including acetylation of their N-termini, amidation of C-termini, modification of sequence or its terminus fragments with unnatural amino acids or other molecules (*e.g.* carbohydrates, lipids), epitope stapling and peptide cyclisation (Fig. 3). For example, cyclization of epitopes has become popular strategy in HIV vaccine development.^24^ Cyclisation of an epitope also restrains its three-dimensional structure and therefore might be used for the assembly of conformational epitopes. Development of consensus epitopes by replacement of some amino acids in an epitope sequence with amino acids presented in corresponding epitope from different strain of the same pathogen, is another interesting strategy for vaccine development. Following this approach, higher antibody titers can be generated against the targeted protein and, more importantly, those antibodies can recognize both pathogen strains used in the design of this epitope.^25^ However, while modification of epitopes may be beneficial, longer peptides still need to be processed to activate desired immune responses.^26^ Recent research by Melief and co-workers has shown that synthetic long peptides induced stronger T-cell activation than the corresponding protein, and rapid antigen processing in APCs was the key factor which allowed enhanced cellular immune responses.^27^ Extensive modification of the peptide epitopes may disrupt such processing resulting in poor performance of vaccines bearing them.

## Adjuvant and delivery systems

The terms *adjuvant* and *delivery system* are no longer mutually exclusive in the field of vaccine development. Both adjuvants and delivery systems can have the potential to stimulate an immune response while simultaneously protecting the antigen degradation and transporting it to the desired tissue. Furthermore, delivery systems are often described as self-adjuvanting or containing a built-in adjuvant. To this end we define delivery systems as technology to administer or transport vaccine components, and adjuvants as agents with the clear ability to enhance the immune response against the antigen of interest. Generally an adjuvant is used in vaccine design as a substitute for the natural “danger signal” that would usually be triggered by infection. While whole pathogen-based vaccine usually have their strong native danger signals, protein, and especially peptide-based vaccines need the help of adjuvant for their efficacy.

Currently there is a wide variety of experimental adjuvants with proven efficacy in the induction of immune responses against peptide.^28^ They are usually the agonists of TLRs, proteins on surface of APCs which recognize PAMPs (Fig. 1); however, the adjuvants following other recognition mechanisms are also discovered.^29^ The choice of an adjuvant (or delivery system) is the second major challenge in peptide vaccine development, shortly after epitope selection.^29^ There is only one widely approved adjutant for human use—alum. However, this adjuvant is a poor immune stimulant, with weak adjuvanting potency for peptide antigens and shortage in ability to stimulate cellular immunity.^30^ Conjugation of the peptide epitope to protein carrier has been used to overcome this problem. For example, the vaccine against Group A *Streptococcus* which entered clinical trials in 2015 was constructed based on the conjugation of a conserved B-cell epitope with diphtheria toxin (DT) protein. DT protein served mainly as a reservoir of T-helper epitopes but also allowed alum to efficiently adjuvant the conjugate.^31^ The presence of a conserved epitope allowed induction of protective humoral immune responses against multiple GAS strains.^31^ A similar conserved-antigen strategy was used to generate broad protection against a wide range of *Pseudomonas aeruginosa* strains.^32^

Other than alum, adjuvants such as squalene-based emulsions AF03, MF59 and AS03, as well as monophosphoryl lipid A-containing AS04 have been also licensed for human application; however, these licenses are country and disease specific.^29^ This poor availability of adjuvant for human vaccine is related mainly to the side effects associated with the use of immune stimulants. For example, one of the most potent inducers of humoral immunity, complete Freund's adjuvant (CFA), is too toxic to use in humans. Even the safety of licenced adjuvant squalene-based MF59 and AS03 came under fire after AS03 was associated with childhood cases of narcolepsy.^33^ Fortunately, continuous development in this field is delivering several new potentially safe adjuvants every year.^34^ While some of these adjuvants are poorly defined bacterial component or large molecules, some of them are relatively simple chemical compounds (*e.g. S*-[2,3-bis(palmitoyloxy)propyl] cysteine, imiquimod, resiquimod, saponins, lipopolysaccharides, imidazoquinolines, polyuridylic acid (polyU)). The latter can be produced using standard chemical approaches. Moreover, large libraries of their derivatives can be synthesised toward identification of the most potent and safe analogues. Beside the development of new ligand for APCs receptors, modification of currently known “natural” adjuvants aiming to reduce their toxicity is a promising approach. One such example is the dephosphorylation of lipid A (TLR-4 ligand derived from bacterial liposaccharide) which significantly reduced its toxicity. The monophosphoryl lipid A in combination with aluminum hydroxide (licensed as AS04) was approved in the GSK human papillomavirus vaccine.

A variety of delivery systems have been used for peptide vaccine development. Such systems should be able to protect protease-sensitive epitope from degradation, and also co-deliver some other vaccine components such as an adjuvant. Such co-delivery can be very important, as the APCs which are taking up peptide antigen, but not adjuvant at the same time, may induce tolerance to the antigen. Moreover, the uptake of peptide antigen and T-helper epitope by the same dendritic cell is also required.^35^ A delivery system can form a depot at the site of injection for prolonged antigen release, or alternatively, can help the vaccine travel through the lymphatic system to reach lymphatic nodes. It may target vaccine to APCs, for example, by aiming mannose receptors presented on them or using dendritic cell-targeting peptides^36^ to boost immune responses. In such cases, the delivery system acts as an adjuvant or can be defined as a self-adjuvanting delivery system.

The use of a delivery system is especially important for vaccine administered by routes other than parenteral. The oral delivery route is most favoured for drugs and vaccines. Such an administration route is convenient, needle-free, economical, and can be performed without the help of trained personnel. However, it is also the most challenging pathway for peptide vaccine delivery. In the gastrointestinal track (GT), the delivery system needs to protect the vaccine against low pH in the stomach, proteolytic enzymes, and bile salts. It should boost the uptake of antigen by the residual immune cells, or at least help the antigen to cross the epithelial membrane, before the vaccine is digested or discharged from the body. Tolerance is other major obstacle associated with oral administration which needs to be overcome. The GT is intended to process nutrients rather than to induce immune responses against it. Liposomes, emulsion, virus-like particles and nanoparticles were widely tested for oral vaccine delivery.^37^ Mucoadhesive polymers such as chitosan are one of the most promising novel platforms for oral delivery.

**Fig. 4:**
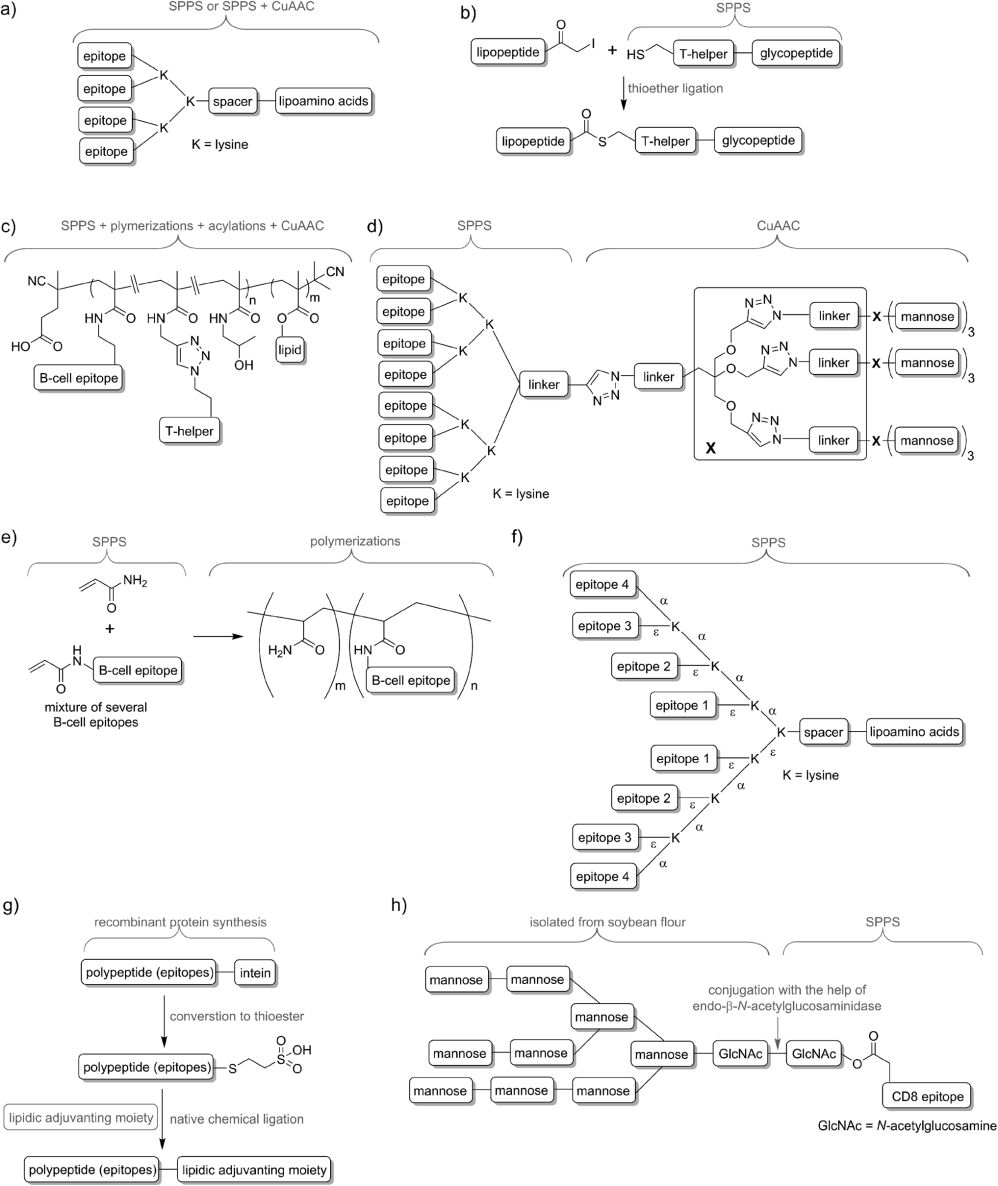
Examples of peptide-based vaccines and synthetic techniques used for their production: (a) lipid core peptide vaccine delivery system (produced by SPPS, occasionally with help of CuAAC); (b) vaccine produced with help of thioether ligation; (c) multicomponent vaccine obtained by polymerization/conjugation approach; (d) asymmetrical dendrimer produced with the help of copper-catalysed azide–alkyne cycloaddition; (e) multi-epitope construct produced by random polymerisation of several acrylate modified B-cell epitopes; (f) multiple different B-cell epitopes incorporated into one entity *via* stepwise SPPS using lysine-based branching; (g) recombinant polyepitope conjugated to adjuvanting moiety with the help of intein and native chemical ligation; (h) glycopeptide-based antigen synthesised in a mixed chemical/enzymatic approach.

Delivery systems are usually physically entrapping the vaccine components in/on the carrier. However, chemical conjugation can be also applied to build more stable delivery platform. For example, lipid core peptides (LCPs) were reported to conjugate several different elements, including lipidic self-adjuvanting moiety (TLR2 ligand), branching moiety for attachments of epitopes and targeting moieties (*e.g.* mannose) and were shown to be able to induce strong cellular and humoral immunity on their own, without the help of adjuvant and other additives (Fig. 4a).^38^ Another system reported by Cai and co-workers combined glycopeptide antigen, T-helper epitope and lipopeptide with the help of thioether ligation (Fig. 4b). The fabricated conjugate was able to elicit a high level of tumour-specific antibodies.^39^ A further interesting conjugation has been reported by Kunz and co-workers (Fig. 4c). In their approach for vaccine development B-cell epitope, T-helper cell epitope and lipid were combined together *via* polymerisation reaction.^40^ The most significant advantageous of all these approaches was construction of vaccine candidates based on single chemical entity. Such strategy should allow better control on vaccine composition and stability, simpler physicochemical characterization, and more easy regulatory approval.

## Multi-epitope-based strategies

In nature, pathogens do not display just a single antigen to the immune system; they are presenting multiple, different antigens and each of them in multiple copies. Unsurprisingly, this natural phenomenon was also adopted in peptide vaccine development. Tam and co-workers demonstrated that multiple antigenic peptide (MAP), a conjugate bearing multiple copies of the same antigen, was able to induce significantly stronger humoral immune responses than un-conjugated peptide epitopes.^41^ In the approach, branched lysines were used as the template to which peptides were attached. Presentation of multiple copies of peptide on such dendritic carriers was expected to protect the peptide from premature degradation and enhanced its recognition by immune cells. The actual mechanism explaining the increased potency of B-cell activation by multimeric antigens over monomeric antigens has only recently been revealed.^42^ Since Tam's early work, vast numbers of vaccine candidates were designed using this approach. An interesting example was reported by Kowalczyk *et al.* ^43^ In their approach which used copper-catalysed azide–alkyne cycloaddition (CuAAC) reaction and lysine branching strategy, asymmetrical dendrimer was produced creating multiple copies of both peptide epitope and carbohydrate targeting APCs (Fig. 4d).

Incorporation of multiple epitopes into vaccine is even more desirable than producing multiple copies of the single epitope. The use of heterovalent vaccine should allow better coverage of natural pathogen antigen diversity, better match the genetic variability of the human immune system, reduce the risk of pathogen escape due to immune pressure (mutational adoption of pathogen similar to that against antibiotics) and may even target several life stages of single or different pathogens. Thus, a simple physical mixture of peptide antigens has been often used for vaccine development. Such mixture-based therapeutic vaccine against HPV has reached advanced clinical trials.^44^ However, the mixture does not assure delivery of the targeted peptide epitopes, T-helper epitopes and adjuvants to the same APCs, and therefore may impair immune responses. To evaluate the importance of conjugation, Boersma and co-workers immunized mice with B cell and T-helper epitopes either conjugated or as a physical mixture.^45^ Covalent linkage between T-helper and B-cell epitopes was required to induce production of epitope-specific antibodies able to recognize the parent protein. Similarly, although generally not required, combination of the epitopes and adjuvant by chemical conjugation was widely reported to increase strength of immune responses.^46,47^ Delivery systems such as liposomes can be used for those purpose^48,49^ but chemical approaches using multiple conjugations are considered even more accurate to ensure the co-delivery of the vaccine components. To produce such constructs, several conjugation techniques can be applied, including native chemical ligation,^50^ maleimide–thiol group reaction,^51^ thioether ligation,^52^ CuAAC reaction,^46^ oxime ligation,^53^ and hydrazone ligation between the aldehyde group and hydrazine (NH_2_NH–) group.^54^ A variety of epitopes were combined in one construct using polymerisation of multiple epitopes derivatised with acryloyl chloride (Fig. 4e).^55^ Multiple B-cell epitopes were also conjugated together *via* stepwise SPPS using lysine-based branching with distinct protective groups on α and ε amine moieties (Fig. 4f).^56^ While many chemical methods can be used for such conjugations, the formation of complex molecules such as glycopeptide epitopes might be synthetically very challenging. These obstacles can be overcome by a combination of chemical methods, with recombinant techniques or enzymatic reactions. Work by Moyle *et al.* resulted in polyvalent vaccine candidate being produced by conjugation of recombinant polypeptide bearing multiple B-cell epitopes to lipidic adjuvanting moiety with the help of native chemical ligation, maleimide, or intein assisted approaches (Fig. 4g).^57,58^ In another study, peptide epitopes bearing fully-defined high-mannose *N*-glycan were synthesised in a mixed chemical/enzymatic approach (Fig. 4h).^59^ This method enabled the production of glycopeptides bearing complex *N*-glycan antigens through a relatively simple short pathway in good yield.

## Nano and micro-technology in vaccines

In general, peptide vaccines need an adjuvant for their efficacy. Adjuvants usually target APCs through TLRs recognition. However, delivery systems targeting APCs designed to mimic pathogen without the involvement of specific receptor recognition are also possible. Antigen uptake by APCs depends on the size, shape, surface, morphological and physicochemical properties. The mechanism of uptake/endocytosis varies depending on the size, and different sizes are preferentially uptaken by different subsets of APCs.^60^ These observations resulted in rapidly increased popularity of nano- and microparticles usage for vaccine delivery in recent years.^61^

It has been demonstrated that nanoparticles can be uptaken preferentially by APCs, especially when they are positively charged. Small nanoparticles (<100 nm) can easily travel to lymph nodes and therefore induce stronger and faster immune responses. Most of the reported studies suggested that 10–50 nm nanoparticles are optimal for induction of humoral and/or cellular immunity;^62–65^ however, the optimal size was different depending on material used for antigen delivery. It also needs to be taken into account that reported sizes of nanoparticles depend on the techniques used to determine their sizes. For example, the size of particles visualised by transmission electron microscopy (dried particles) may significantly differ from the perceived hydrodynamic size in solution as measured by dynamic light scattering.

In contrast to small nanoparticles which are easily trafficking in lymphatic system, large nanoparticles and microparticles can induce a strong immune response due to depot effect (retention of the formulation and slow antigen release at the injection site). Perrie and co-workers demonstrated that liposomes with longer retention at injection site induced stronger Th1 immune response.^66^ In addition, a particle-based delivery system may allow antigen cross-presentation toward inducing cellular immunity (for example, against cancer).^67^ Particles, similar to other delivery systems, can also trigger stronger immune responses due to the presence of multiple copies of epitope on their surface and protection of peptide against enzymatic degradation. Interestingly, shape (spherical over cylindrical) and enhanced hydrophobicity of the particles was also reported as factors influencing immune system activation.^68,69^

Several delivery platforms have been used for particle-based vaccine development including polymers, lipids (including liposomes), inorganic particles and even carbon nanotubes.^70^ Some adjuvants are also reported to act as particles for vaccine delivery (*e.g.* saponin-based ISCOM which forms 40 nm nanoparticles).^71^ In particular, polymers have been widely studied for peptide delivery. Among these, the most widely investigated were biodegradable polymers, including poly(d,l-lactic-*co*-glycolide),^72^ chitosan,^73^ and poly glutamic acid;^74^ however non-biodegradable polymers such as polystyrene were also considered.^75^ The most popular production pathway of particles includes pre-assembly of the polymer to form beads followed by antigen entrapment; however, conjugation of polymer to peptide following the self-assembly process is also possible.^40,76^ Biodegradable polymers provide, in general, a better safety profile, while non-biodegradables are expected to form more stable particles. In both cases, one of the major drawbacks is the polydispersity of polymeric material. It might be difficult to obtain pharmaceutical-grade reproducibility of the nanomaterial (nano-vaccine) when one of the main components (polymer) has its own polydispersity range. This property is clearly disadvantage of polymers but do not halt their use for pharmaceutical purposes.

Polyethylene glycol (PEG) is one of the most commonly used polymers in the medical sciences and several pharmaceutical products incorporating this polymer have been approved by the FDA. PEG is biocompatible, inert polymer and can protect a drug from degradation and elimination *in vivo*. PEG was also used for vaccine delivery; however, while it can allow prolonged blood circulation time of the antigen, it greatly reduced the ability of the immune system to recognise such material. Cui and co-workers use this phenomenon for targeted delivery of PLGA nanoparticles to specific subsets of macrophages.^77^ They produced mannosylated particles covered by PEG which was selectively hydrolysed off (unhidden) at the tumour site. Therefore the particles were taken up preferentially by tumour-associated macrophages rather than normal macrophages.

Inorganic particles may serve as a relatively inert delivery system; however, the lack of biodegradability might pose safety considerations similar to those of non-biodegradable polymers. Materials such as aluminum oxide,^78^ gold^65^ and calcium phosphate^79^ have been used for peptide-based vaccine delivery. In contrast, lipid-based particles are usually biocompatible and biodegradable. They not only are able to assemble into micelles or liposomes, they also might be recognised by TLRs (especially TLR2 and 4 which naturally recognize lipidic ligands).^80^ For nanoparticle peptide-based vaccine delivery, lipids are usually used as adjuvanting moieties (*e.g.* LCP,^81^ Pam2Cys^82^) and/or hydrophobic cores which allow the self-assembly of peptide epitopes conjugated to them. Self-assembled lipid–peptide conjugates have shown the ability to induce both humoral and cellular immune responses.^83,84^ Finally, self-assembling constructs exclusively composed of peptides are also possible. Such example was reported by Collier and co-workers.^85^ In their delivery system, peptide adopting β-sheet conformation was conjugated to the peptide epitope allowing the product to self-assemble into nanofibers. This construct, built only of peptides, was able to induce immune responses against incorporated epitope without the help of any adjuvant.

While the major advantage of a nanoparticle-based delivery system is its ability to induce immune responses without the help of adjuvant, co-formulation of nanoparticles with immunostimulant might induce even stronger immune responses. This combination treatment may also result in a substantial reduction of the adjuvant quantity required to boost immunity and therefore reduce any undesired side effects associated with the adjuvant. This dose reduction can be as high as 100-fold compared to the originally required quantity.^86^

## Current stand and future perspective

No peptide-based vaccines are currently available on the market. However, a large number of peptide vaccines have recently reached clinical trials.^87^ The development of peptide-based vaccines is a relatively new area in the vaccination world, thus the surge of interest in this strategy should not be unexpected. Despite its novelty and promise, this strategy is vulnerable to our limited knowledge. The current understanding of the immune system and pathogenesis has improved tremendously in comparison to that of the early years of vaccination, but the picture is still not complete. While whole pathogen-based vaccine might be designed, produced and successfully applied without an in-depth understanding of human immunity, the use of minimal antigenic component (*i.e.* peptide) for vaccine development needs extensive knowledge of the immune system. In addition, as proper selection of peptide antigen is crucial for the vaccine efficacy, the knowledge about the pathogen's life cycle, including host/cell entry and survival mechanism, is critical. Consequently, the vaccine could be designed to induce immune responses that target key points in pathogen invasion, for example a protein that is responsible for bacterial adhesion to the surface of the host cell.

Vaccine composed of whole pathogen most likely carries the “danger signal” and therefore the use of adjuvant is often not required. In the case of peptide-based vaccine, adjuvant (or self-adjuvanting delivery system) is crucial. However, once again, general knowledge about adjuvants is limited. The mechanism of action of the only generally approved adjuvant, alum, is still not fully understood and multiple mechanisms are being reported.^29^ Fortunately the immunology is one of the most quickly advancing fields of research, including both the enhancement of general knowledge of the immune system as well as the understanding of the relationship of the pathogen with the immune system. For example, new conserved neutralising epitopes have been identified in HIV-1 and influenza, thereby opening the door for design of universal vaccine against AIDS and flu.^88–90^

As mentioned several time in this review, one of the major issues in vaccine development is the identification of the potent and safe immune stimulators. Nanotechnology can come to the rescue, offering not only a self-adjuvanting delivery system, but also a role in the reduction of toxicity of currently studied experimental adjuvant. Reactogenicity of an adjuvant can be minimised by its targeting delivery to APCs and the dose reduction. For example, nanoparticles-bearing CpG and peptide epitope were more effective in induction cellular immunity than a few-fold higher dose of peptide and adjuvant delivered without nanoparticles.^91^ A similar ability to reduce the amount of toxic adjuvant was reported for liposomal formulation of poly(I:C) adjuvant.^92^ Interestingly, while the size is a well-proven factor influencing immune responses; it is rather difficult to identify a single optimal size for all vaccine formulations. It is an intriguing idea to use a well-defined mixture of particles with different sizes to stimulate optimal immune responses. For example, a depot effect can be created by microparticles, while at the same time; the identical antigen in nanoparticle form can be delivered directly to lymph nodes.

There are also some dangers associated with the use of nanotechnology. Besides the obvious ones related to the potential toxicity of nanoparticles, especially those positively charged,^93,94^ the use of biodegradable polymers may result in unexpected complications. Whereas, from the toxicological point of view, biodegradable materials are favoured, the presence of such components in the nanoparticles may change the vaccine's properties during administration. Resultant size deviations (due to degradation) of nanoparticles may change their excretion speed and immunological properties. Moreover, such changes may differ significantly between the animal and human models. Consequently, in the early development process of peptide-based vaccines, not only general efficacy should be taken into account, but also the properties typically associated with drugs rather than vaccines (*i.e.* there is a need to extensively understanding of pharmacokinetics).

The other danger derives from epitope recognition and pathogen escape sides. While the use of highly conserved single epitope might not be enough to guarantee a high efficacy of the vaccine in the widespread human population, the use of the multi-epitope approach might be tricky. The application of modern organic chemistry may permit the conjugation of several different epitopes, adjuvanting and targeting moieties into one construct (Fig. 4). However, similar to approaches using total synthesis of natural products for drug purposes, the final product might be too laborious to produce, difficult to scale up, or simply too expensive. Thus, a physical mixture of peptides can be used with appropriate formulation to assure co-delivery of vaccine components. Chemical conjugation approaches based on polymer chemistry (polymerization of multiple different components) also seem to be promising; however, polydispersity and lack of full definition of such products need to be taken into account. Another option includes the formation of a simple particle-based unit incorporating all the necessary elements (*e.g.* single B-cell epitope, T-helper epitope, an adjuvant) and then mixing the different units to achieve the desired vaccine formulation.

On the bright side, we can expect a peptide-based vaccine available on the market soon. This assumption is not only based on hundreds of clinical trials which have been performed to this point in time,^87^ but also one unique future of peptide-based vaccines. This is the fact that such vaccines are the perfect material for the induction of cellular immunity. In general, the cellular immune system does not recognise the pathogen itself or its surface as humoral immunity does. Cellular immune system recognizes specific peptide epitopes displayed by the MHC I protein on the surface of human cells. Indeed, one of the most studied targets for peptide-based vaccines is cancer, where the whole cell-based approach is obviously pointless, and protein-based approaches that predominantly target humoral immunity demonstrate limited ability to eradicate any tumour. Therefore, as expected, a large number of clinical trials on cancer immunotherapy are now exploring the efficacy of peptides to serve as antigens in therapeutic anticancer vaccines.^95^ Of course, promising properties of peptide-based immunotherapies in targeting cellular immunity do not disqualify them to be developed as standard prophylactic vaccines targeting humoral immune responses.

## Conclusion

Classical vaccination using whole organisms is usually cheap and despite the drawbacks associated with production difficulties or safety, whole pathogen-based vaccines will not disappear from the market any time soon. However, during the same time, it can be expected that highly defined vaccines based on small antigens will start to slowly replace the whole pathogen approach. Vaccines entirely produced *via* chemical synthesis might be especially attractive as they evade the use of any cell-derived material or biological processes for their production. Therefore, their purity can be highly controlled, in exactly the same manner as has been established for classical drugs. Further advances in organic and polymer chemistry should reduce the cost of synthetic vaccine production. A better understanding of the immune system should allow for a more “intelligent design” of peptide-based antigens, delivery system, and adjuvants required for the vaccine efficacy in the induction of immune responses. Taking into account the reduced side-effects and improved stability of peptide-based vaccines as well as compatibility with the therapeutic approach, we can expect a major breakthrough in the field, sooner rather than later.
